# Protective effect of *Tisochrysis lutea* on dry eye syndrome via NF-κB inhibition

**DOI:** 10.1038/s41598-022-23545-7

**Published:** 2022-11-15

**Authors:** Sung-Chul Hong, Hyung Seok Yu, Jin-Woo Kim, Eun Ha Lee, Cheol-Ho Pan, Kwang Won Hong, Jin-Chul Kim

**Affiliations:** 1 Smart Farm Research Center, Korea Institute of Science and Technology (KIST), Gangneung, 25451 Republic of Korea; 2 Natural Product Research Center, Korea Institute of Science and Technology (KIST), Gangneung, 25451 Republic of Korea; 3 Department of Food Science and Biotechnology of Animal Resources, Konkuk University, Seoul, 05029 Republic of Korea; 4 Department of Agricultural Biotechnology, Seoul National University, Seoul, 08826 Republic of Korea; 5 Natural Product Informatics Research Center, Korea Institute of Science and Technology (KIST), Gangneung, 25451 Republic of Korea; 6 Microalgae Ask Us Co., Ltd, Gangneung, 25441 Republic of Korea

**Keywords:** Nutritional supplements, Corneal diseases, Nutrition

## Abstract

Dry eye syndrome (DES) affects the cornea, causes pain and hypersensitivity to light. Although inflammation and endoplasmic reticulum stress are known to be involved, the detailed mechanisms remain unknown. DES is characterized by a decrease in corneal thickness, tear volume, and lacrimal gland size, and damage to corneal cells. *Tisochrysis lutea* is a microalga that has been shown to reduce immune factors. Therefore, we hypothesized that *T. lutea* could ameliorate DES. We investigated the role of *T. lutea* in scopolamine-induced DES in BALB/c mice. Oral administration of *T. lutea* increased corneal thickness, tear volume, and size of the corneal cells, and reduced damage to the corneal cells. Furthermore, treatment of ARPE-19 human retinal pigmented epithelial cells with *T. lutea* reduced expression of the inflammatory factor, NF-κB, MAPK, and AKT. *T. lutea* may be used therapeutically to reduce the symptoms of DES.

## Introduction

The prevalence of dry eye syndrome (DES) has increased worldwide, affecting around 50% of all adults^1^. DES is a multifactorial ocular surface disease characterized by tear film instability and hyperosmolarity, ocular surface inflammation and damage, neurosensory abnormalities (Tear Film and Ocular Surface International Dry Eye WorkShop II)^2^. DES is frequently associated with symptoms of ocular discomfort, such as foreign body sensation, dryness, irritation, burning, and light sensitivity, and has a significant detrimental effect on quality of life^3,4^. It is also strongly associated with anxiety and depression^5^. Age, sex, ethnicity, medical conditions (e.g., connective tissue disease, Sjögren’s syndrome, androgen deficiency, and diabetes), contact lens use, use of systemic medications (e.g., estrogen replacement therapy, antihistamines, anxiolytics, antidepressants, and diuretics), and environmental conditions (e.g., computer usage, low ambient humidity, and air pollution) have all been identified as risk factors for DES^6–9^.

Both pharmacologic and non-pharmacologic therapeutic options are available for DES control and treatment^10,11^. The first-line treatment is the use of artificial tear drops to supplement the tear film; however, this approach only provides temporary symptomatic relief, and up to two-thirds of DES patients continue to experience symptoms despite treatment^12^. Other treatment options include tear retention devices or procedures (e.g., punctual occlusion, moisture-conserving spectacles, and contact lenses), tear stimulation (e.g., intranasal neurogenic tear stimulation and vectored thermal pulsation), pharmacologic tear stimulation (i.e., secretagogues), and biological tear substitutes (e.g., serum and salivary gland aureoles) depending on the severity of the disease^13–16^. In addition to medical treatment, the consumption of non-drug-treated functional foods may be beneficial in DES treatment. Polyunsaturated fatty acids (PUFAs), which contain two or more double bonds in their hydrocarbon chain, have been suggested to alleviate DES symptoms^17,18^. Omega-3 (ω-3) and omega-6 (ω-6) PUFAs, which have their first double bonds located three or six carbons from the methyl end, respectively, are the most well-known. Humans cannot synthesize ω-3 or ω-6 PUFAs in vivo; therefore, these fatty acids must be obtained via the diet or supplementation.

The ω-3 PUFAs, eicosapentaenoic acid (EPA), docosahexaenoic acid (DHA), and α-linolenic acid (ALA), are frequently used as food supplements^19,20^. While short-chain ω-3 PUFAs, such as ALA, are abundant in certain vegetable oils (flaxseed and canola) and terrestrial plants (walnuts and chia seeds)^21,22^, long-chain ω-3 PUFAs, such as EPA and DHA, are primarily derived from fish oils (from salmon, mackerel, anchovies, and sardines) and, to a lesser extent, other marine sources (oysters, mussels, and prawns)^23–25^. ω-6 PUFAs, such as arachidonic acid and linoleic acid, are found in various vegetable oils (corn, safflower, and sunflower seed) as well as meats and other animal products. Short-chain ω-3 PUFAs can be converted to long-chain ω-3 PUFAs in the body; however, the efficiency of conversion varies with age^26^. The ratio of ω-3 to ω-6 PUFAs in vivo may be determined by the individual’s inflammatory status^27,28^. Derivatives of EPA, which is an ω-6 PUFA, are predominantly proinflammatory (prostaglandin-E2, thromboxane-A2, and leukotriene-B4), whereas metabolism of long-chain ω-3 PUFAs produces anti-inflammatory eicosanoids (e.g., resolvins and protectins), which can inhibit the production of proinflammatory mediators such as interleukin-1 (IL-1) and tumor necrosis factor (TNF-α)^29,30^. These mechanisms have been linked to ocular surface damage and tear film quality in individuals with DES. Therefore, various natural products have been examined to determine whether they contain sufficient PUFA to mitigate the effects of DES.

Interestingly, microalgae are revealed to be high in PUFA. Especially, under certain conditions, the lipid content of microalgae can range from 30%–70% of the cell dry weight^31^. Microalgae lipids are primarily composed of saturated and monounsaturated C14–C20 fatty acids^32^. Furthermore, some microalgae species can synthesize significant amounts of EPA and DHA. *Nannochloropsis* spp., *Botryococcus* spp., *Scenedesmus* spp., *Spirulina* spp., *Chlorella* spp., *Dunaliella* spp., *Phaeodactylum tricornutum*, *Isochrysis galbana*, *Monodus subterraneus*, *Tetraselmis* spp., and *Chlamydomonas reinhardtii* are lipid-rich microalgae species with high productivity (20–200 mg/L/day) and are widely cultivated as food sources^33,34^. Highly controlled environments of cultivation tanks (ponds or photobioreactors) enable the microalgae to produce sufficient amounts of ω-3 PUFAs and provide an alternative to fish oils^35,36^.

The present study aimed to confirm the presence of PUFAs in *Tisochrysis lutea*, which is a novel microalga that is not well understood. Specifically, we examined the effect of PUFAs on DES using an animal model and investigated the use of *T. lutea* as a functional food material to ameliorate DES. We also investigated the mechanism of action involved in DES improvement to maximize its potential use in future.

## Results

### Effect of *T. lutea* on tear production

**Figure 1:**
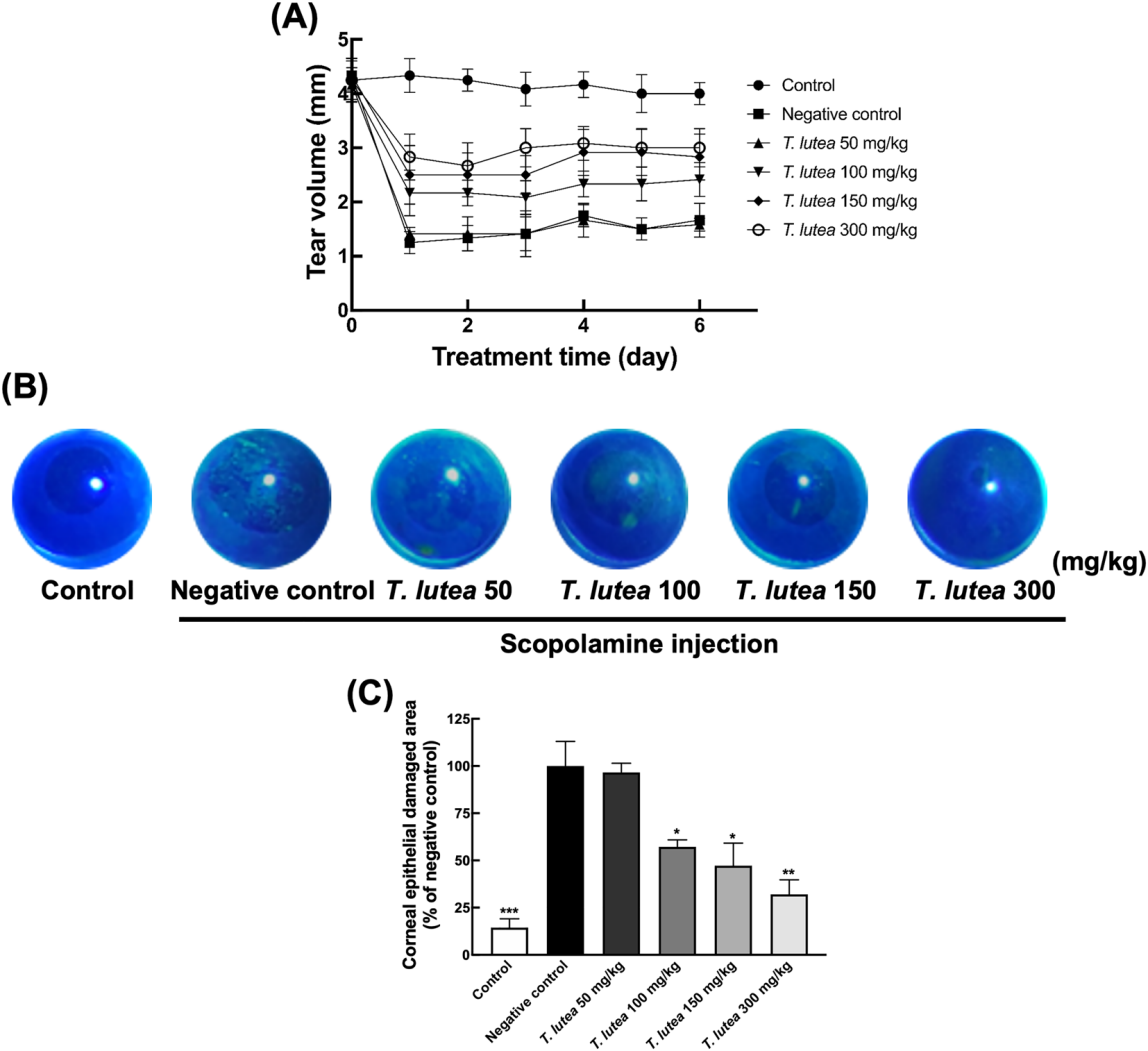
Effects of *T. lutea* on eye damage and tear production. Dry eye (DE) was induced by scopolamine injection, and *T. lutea* was administered at concentrations of 0 (control; CON, without DE induced by scopolamine injection), 50 (*T. lutea* 50), 100 (*T. lutea* 100), 150 (*T. lutea* 150), or 300 (AKE 300) mg/kg. (**A**) Tear volume was measured using Schimer’s test. (**B**) Representative images of corneal fluorescein staining. (**C**) Quantitative analysis of images in (**B**). Each treatment group consisted of 7 mice (n = 14 eyes). Data are represented as mean ± SEM (error bar). *p < 0.05, **p < 0.01, and ***p < 0.001 versus negative control. Data were analyzed statistically using one-way ANOVA followed by Tukey’s post hoc test.

Scopolamine was injected subcutaneously into 6-week-old BALB/c mice twice daily for two weeks to induce DES. *T. lutea* was administered orally at a constant concentration daily and the eye size of five mice was measured for seven days to evaluate its effects on DES. The number of tear volume decreased by approximately 3 mm per 1 mm in the DES-induced group compared with the control group (Fig. 1A). On the other hand, the volume of tears produced in the *T. lutea* treatment group increased significantly at a concentration.

### Effect of *T. lutea* on eye damage

When DES is provoked, the blinks increase to stimulate tear production. However, there is no increase in tear production due to DES, and cornea surface scratches are caused by frequent blinking. The degree of corneal damage was determined to confirm such phenomenological findings. The degree of corneal damage was quantified by performing fluorescent staining in a dark room, in which the cornea was illuminated with blue light to confirm the damaged surface. We confirmed that the degree of corneal damage decreased as the *T. lutea* treatment concentration increased (Fig. 1B,C).

### Histological alterations of the corneal epithelial following *T. lutea* treatment in the DES mouse model

**Figure 2:**
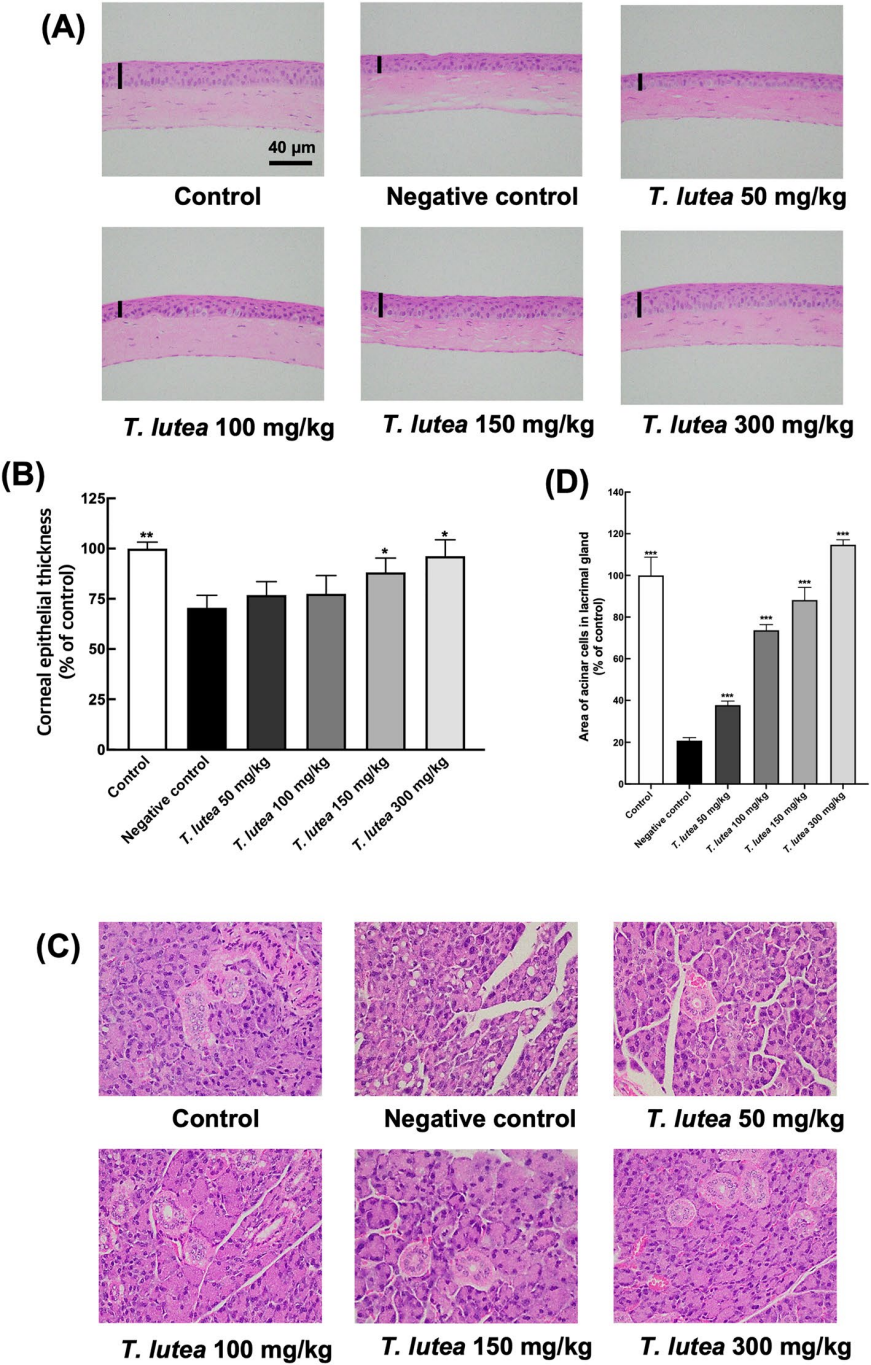
Corneal and lacrimal gland tissue changes following *T. lutea* ingestion. (**A**) H&E staining of whole-eye sections to demonstrate corneal epithelial thickness. (**B**) Representative graph of corneal thickness. (**C**) H&E staining of lacrimal gland tissue sections to demonstrate lymphocyte infiltration. (**D**) Quantity of acinar cell area in lacrimal gland tissues. Each treatment group consisted of 7 mice (n = 14 eyes and n = 7 lacrimal gland tissues). Data are represented as mean ± SEM (error bar). *p < 0.05, **p < 0.01, and ***p < 0.001 versus negative control. Data were analyzed statistically using one-way ANOVA followed by Tukey’s post hoc test.

Induction of DES may lead to thinning of the corneal tissue due to corneal damage. H&E staining was used to observe changes in the corneal tissue. Sectioning revealed that the corneal tissue was significantly more delicate in the DES-induced group than in the control group (Fig. 2A,B). The corneal thickness increased in a concentration-dependent manner in the *T. lutea*-treated group.

### Histological alterations of the lacrimal glands following *T. lutea* treatment in the DES mouse model

Lacrimal tissue changes were examined in scopolamine-induced DES mice with to ascertain the cause of the changes in eye volume. After confirming the degree of linear cell atrophy and the presence or absence of cell gaps in the lacrimal gland tissue, the acinar cells in the control group showed a uniform size and almost no cell gaps. However, in the DES group, cells within the lacrimal gland tissue were atrophied and numerous cell gaps were present (Fig. 2C,D). Mice treated with low concentrations of *T. lutea* showed linear cell atrophy and cell gaps occurred with the same frequency as the mice treated with DES, but the group treated with high concentrations of 150 and 300 mg/kg of *T. lutea* showed reduced atrophic cell gap of the acinar cells and increased acinar cells. In the DES mouse model, the lacrimal gland tissue was destroyed, but was restored in response to *T. lutea* intake.

### Effect of *T. lutea* on cell viability

Following our evaluation of the ameliorative effect of *T. lutea* on DES in advanced animal models, we investigated the anti-inflammatory effects and mechanisms in ARPE-19 cells to determine whether *T. lutea* could reduce corneal damage and alleviate inflammation. MTT assay was used to determine toxicity with dose-escalation testing to determine whether the anti-inflammatory properties of *T. lutea* were due to cytotoxicity (Fig. S1). *T. lutea* had a detrimental effect on cell viability at concentrations up to 2000 μg/mL. Therefore, since cytotoxicity occurred at concentrations ≥ 125 μg/mL, the effect of enhancing anti-inflammatory in ARPE-19 cells was evaluated at concentrations ≤ 100 μg/mL.

### *T. lutea* inhibited IκB-α degradation and NF-κB activation

Exposure of ARPE-19 cells to physiological and chemical stress leads to the production of various proinflammatory cytokines and initiates an inflammatory response. Therefore, we investigated the effect of postinflammatory *T. lutea* on the inflammatory mechanism of cells using the proinflammatory cytokine, TNF-α.

**Figure 3:**
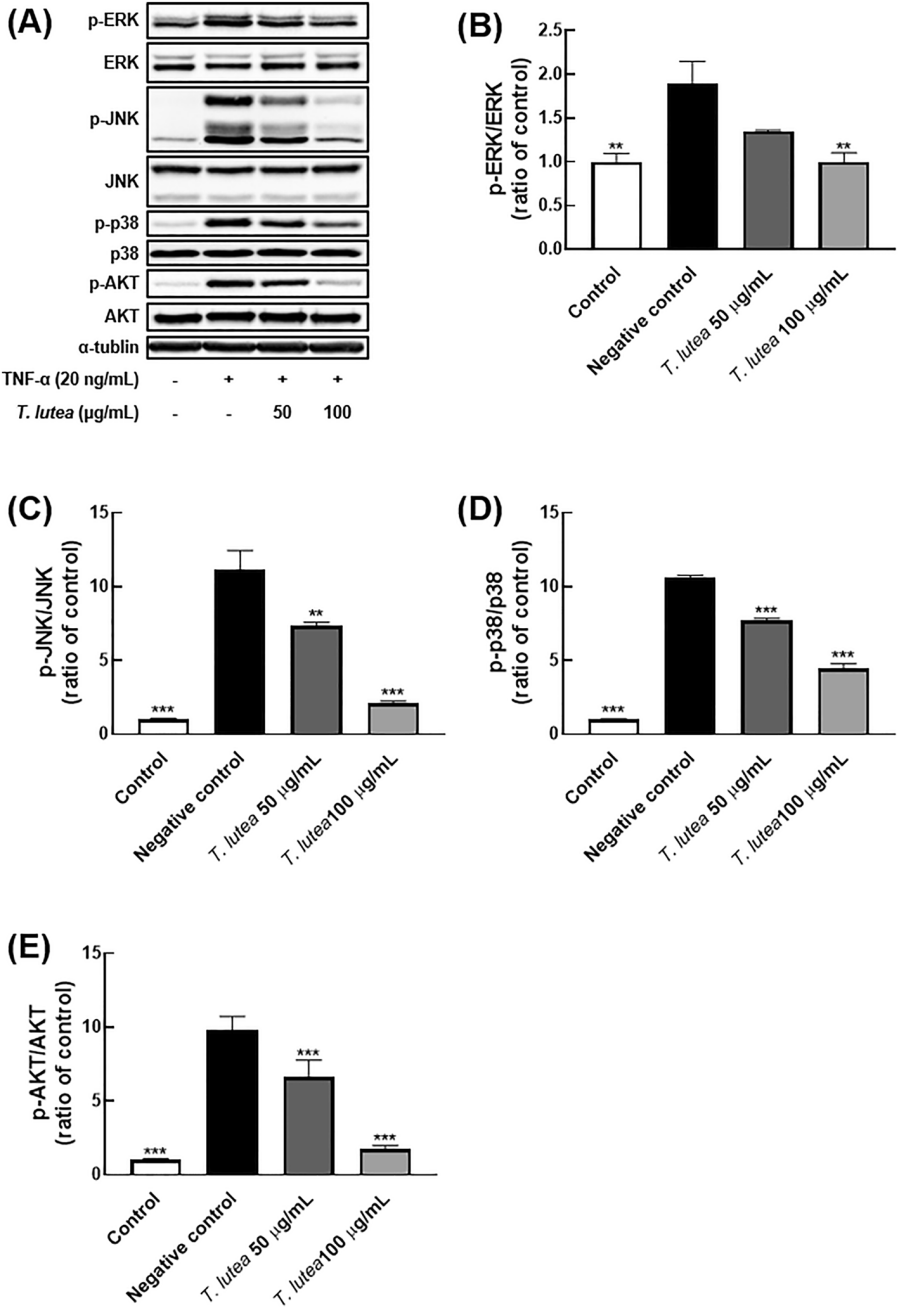
Comparative analysis of MAPK and AKT protein expression levels following *T. lutea* treatment in ARPE-19 cells following TNF-α-induced inflammation. (**A**) Western blot comparison of protein expression. Quantitative comparative analysis of phosphorylation of (**B**) ERK, (**C**) JNK, (**D**) p-38, and (**E**) AKT proteins. **p < 0.01 and ***p < 0.001 versus negative control. Data were analyzed statistically using one-way ANOVA followed by Tukey’s post hoc test.

We focused on the regulatory function of NF-κB signaling to assess the anti-inflammatory activity of *T. lutea*. It is well established that NF-κB signaling is involved in the production of proinflammatory factors. External stimuli, such as TNF-α, promote IκB-α degradation, nuclear p65 metastasis, and nuclear p65 binding to proinflammatory factor-related DNA to express chronic proinflammatory factors^37^. Additionally, in addition to regulating proinflammatory factors, it is critical to control the signal transduction associated with the inflammatory response to improve inflammatory diseases. We examined IκB-α degradation and p65 nuclear metastasis suppression to determine the inhibitory activity of *T. lutea* against NF-κB signaling. TNF-α induced degradation of IκB-α in cells treated with *T. lutea*, whereas *T. lutea* significantly suppressed degradation of IκB-α (Fig. 3). Additionally, TNF-α induced nuclear metastasis of p65, whereas *T. lutea* inhibited TNF-α-induced nuclear metastasis of p65 in a concentration-dependent manner.

### *T. lutea* suppressed AKT and mitogen-activated protein kinase (MAPK) activation

**Figure 4:**
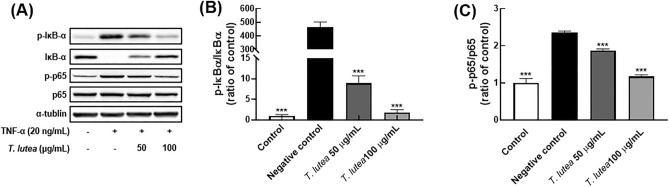
Comparative analysis of NF-κB protein expression in ARPE-19 cells following TNF-α-induced inflammation. (**A**) Western blot comparison of protein expression. Quantitative comparison of phosphorylation of (**B**) IκB-α and (**C**) p65 proteins. ***p < 0.001 versus negative control. Data were analyzed statistically using one-way ANOVA followed by Tukey’s post hoc test.

Our findings showing the NF-κB-suppressive efficacy and associated cytokine changes confirmed the precise NF-κB-suppressive mechanism. Expression of MAPK and AKT were confirmed as related cytokines. In cells not treated with *T. lutea*, phosphorylation was induced by TNF-α and MAPK-related proteins, ERK, JNK, p38, and AKT. On the other hand, *T. lutea* treatment resulted in the phosphorylation of MAPK-related proteins and AKT and levels of TNF-α decreased proportionally to the concentration to *T. lutea*. Therefore, it was concluded *T. lutea* suppresses NF-κB activation via regulation of AKT signaling (Fig. 4).

### PUFA profiling of *T. lutea*

We analyzed the components of *T. lutea* and showed that it decreased tear volume due to lacrimal gland tissue destruction and had a regenerative effect on corneal damage. We used GC analysis to quantify the PUFA levels in *T. lutea*. PUFAs are effective in the treatment of DES and have anti-inflammatory properties. We confirmed that *T. lutea* contained 14 different types of PUFA (Table 1 and Fig. S5), among which DHA was the primary component.

**Table 1:** Fatty acid composition of *T. lutea* determined using GC-FID.

Peak no.	RT (min)	Name		Contents (mg/g)
1	18.706	Myristic acid	C14:0	10.99 ± 0.52
2	19.970	Myristoleic acid	C14:1	0.2 ± 0.01
3	20.373	Pentadecylic acid	C15:0	0.13 ± 0.01
4	22.103	Palmitic acid	C16:0	5.54 ± 0.38
5	23.182	Palmitoleic acid	C16:1	2.02 ± 0.11
6	23.822	Margaric acid	C17:0	0.18 ± 0.01
7	24.809	*cis*-10-Pentadecenoic acid	C15:1	0.61 ± 0.03
8	25.377	Stearic acid	C16:1	0.03 ± 0.01
9	26.374	Oleic acid	*cis*-C18:1	2.5 ± 0.18
10	27.865	11-Eicosenoic acid	C20:1	2.95 ± 0.18
11	28.989	Arachidic acid	C20:0	0.11 ± 0.01
12	29.731	Linolenic acid	C18:3	5.44 ± 0.28
13	36.681	*cis*-5,8,11,14,17-Eicosapentaenoic acid (EPA)	C20:5	0.46 ± 0.03
14	42.438	*cis*-4,7,10,13,16,19-Docosahexaenoic acid (DHA)	C22:6	8.65 ± 0.52

## Discussion

Changes in dietary habits toward increased beef, pork, and chicken consumption have led to a decrease in ω-3 PUFA consumption. The typical dietary ratio of ω-6 to ω-3 is around 15:1 or 16:1, but a healthy ω-6 to ω-3 ratio in the diet is between 1:1 and 4:1^38^. Therefore, the source of ω-3 PUFAs is critical for those with a dietary structure that requires the consumption of ω-3 PUFAs and microalgae are one of the best candidates. Recent interest in microalgae as a novel food source has increased in Europe^39^. Few microalgae have been demonstrated to be substantially functional. Therefore, the present study investigated the treatment of DES using *T. lutea*, a microalga with unknown functions.

PUFAs component analysis of *T. lutea* revealed that DHA, an ω-3 fatty acid, was the primary component. The ω-6 to ω-3 ratio of *T. lutea* is approximately 1:5, which contributes significantly to improved eating habits and it was reported that it could be used as a functional material. Various PUFAs found in *T. lutea*, such as DHA and EPA, have been shown to alleviate inflammation and improve DES.

Ingestion of *T. lutea* was reported to improve DES after inducing lacrimal gland dysfunction using scopolamine and inducing DES in an animal model. Subcutaneous administration of scopolamine induces DES via the lacrimal gland due to increased immune cell infiltration followed by increased levels of proinflammatory gene expression levels. As the volume of tears in the eye decreases, frequent blinking induces inflammation of the corneal surface due to cell damage. To confirm the ameliorative effect of *T. lutea* on DES during the induction process, a mouse model was used to determine the seven times physical measurement during the ingestion period, degree of corneal damage, and corneal tissue thickness. We confirmed the changes in lacrimal gland tissues and concluded that ingesting *T. lutea* improved the state of cell infiltration in the lacrimal gland in a concentration-dependent manner (Fig. S2). Additionally, eye tear volume increased concentration-dependently in response to lacrimal gland tissues restoration. Ingestion of *T. lutea* also improved the degree of corneal damage and the thickness of corneal tissue in a concentration-dependent manner.

Previous studies have suggested that these changes in the lacrimal gland result from an immune response^40^. Scopolamine was also shown to alter the levels of the transcription factors, IL-17, IL-23, IL-6, and TNF-α, which are lacrimal gland immune factors. ARPE-19 cells were used to confirm these effects. The improvement of intracellular immune function was evaluated using *T. lutea*. at concentrations of 50 and 100 μg/mL, which are below the cytotoxic levels of *T. lutea*. Enhanced immune function was demonstrated by changes in immune-related factors, such as NF-κB, MAPK, and AKT. External pathway open-associated molecular patterns, TNF receptors, Toll-like receptors, and IL-1 receptors mediate NF-κB in cells^41,42^. When stimulated, the IKK complex binds to and inhibits activation of the NF-κB dimer, which dissociates and activates IkB^43^. The IkB-separated NF-κB dimer crosses the nuclear envelope (typically the p50–p65 dimer) and enters the nucleus, where it binds to DNA and activates gene transcription. We induced inflammation in ARPE-19 using TNF receptor-stimulating TNF-α to confirm the regulation of NF-κB activity that results in an inflammatory response via these pathways by *T. lutea*. Phosphorylation of IκB-α and p65, which are NF-κB active proteins, decreased concentration-dependently. *T. lutea* has been shown to inhibit NF-κB activity by simultaneously regulating both IκB-α and p65, which are NF-κB subunits that regulate gene expression of protein complexes and proinflammatory cytokines.

We also evaluated *T. lutea* inflammatory activity suppression for an upper mechanism of the IκB-α and p65 subunits. AKT is involved in the phosphorylation of proteins, such as caspase-9 and GSK-3B, and the regulation of metabolism and transmission of signals involved in cell life and death^44^. AKT modulates NF-κB activity, thereby affecting expression levels of the IKK complex, which is composed of IKK-α, β, and γ^45^. The present study confirmed that AKT phosphorylation decreased in a concentration-dependent manner in the *T. lutea*-treated group, which influenced the decrease in the IKK complex expression levels. MAPK is also involved in cell growth and division, stress, and cytokine regulation^46^. Application of an external stimulus activates the signal transduction pathway mediated by ERK, JNK, and p38 intracellularly, resulting in cell morphological changes and cytokine transcription. Phosphorylation of p38 MAPKs induces the activity of p65, an NF-κB activator^47^. Examining the changes in the activity of these MAPKs revealed that *T. lutea* decreased levels of ERK, JNK, and p38, which are MAPKs constituent proteins, in a concentration-dependent manner. Our findings suggest that *T. lutea* inhibits NF-κB activity by inhibiting active MAPKs and AKT pathways activated by TNF-α, thereby exerting an anti-inflammatory effect.

## Conclusion

*T. lutea* improved scopolamine-induced DES and restored cell atrophy caused by inflammation of the lacrimal gland. In particular, corneal damage through increased tear volume was reduced via restoration of the lacrimal gland cells. These anti-inflammatory effects in ARPE-19 cells confirmed that *T. lutea* plays a role in inhibiting NF-κB by suppressing the activity of AKT and significant MAPK proteins. It can be inferred that the improvement effect of *T. lutea* in DES is due to a large amount of PUFAs, in particular, DHA, which is the main component of *T. lutea*. The findings of the present study confirmed the ameliorating effect and mechanisms of *T. lutea* on DES and showed that diets rich in ω-3 PUFAs that can improve eating habits. Therefore, our findings indicate that *T. lutea* can be used as a healthy functional food material.

## Materials and methods

### Materials

#### In vivo/in vitro materials

*T. lutea* was obtained from Microalgae ask us Corp. (Gangneung, Korea). Scopolamine, fluorescein, and hematoxylin and eosin (H&E) were purchased from Sigma-Aldrich (St. Louis, MO, USA) for animal experiments. Dulbecco’s Modified Eagle’s Medium (DMEM):F12, Dulbecco’s phosphate-buffered saline (DPBS), fetal bovine serum (FBS), and 0.25% trypsin–EDTA solution were obtained from Life Technologies (Carlsbad, CA, USA). Antibiotic solution, comprising 10,000 U/mL penicillin and 10,000 μg/mL streptomycin, was purchased from Hyclone Laboratories Inc. (South Logan, UT, USA). Recombinant human TNF-α was obtained from R&D systems (Minneapolis, MN, USA). Primary antibodies including anti-extracellular signal-regulated kinase (ERK), anti-c-jun N-terminal kinase (JNK), anti-p38, anti-α-tubulin, and anti-β-actin were purchased from Santa Cruz Biotechnology (Santa Cruz, CA, USA). Other primary antibodies and horseradish peroxidase (HRP)-conjugated secondary antibodies were obtained from Cell Signaling Technology, Inc (Beverly, MA, USA). Chemicals including 3–4,5-dimethylthiazol-2-yl)-2,5-diphenyltetrazolium bromide (MTT) were purchased from Sigma-Aldrich (St. Louis, MO, USA).

### Chemical analysis materials

PUFAs were identified using a solution containing 37 PUFA standard substances, which was purchased from Sigma-Aldrich (St. Louis, MO, USA). Valeric acid was obtained from Sigma-Aldrich (St. Louis, MO, USA) for use as an internal standard. Solvents, including boron trifluoride methanol solution, *n*-hexane, Na_2_SO_4_, CH_3_Cl, MeOH, and NaCl, used to extract PUFAs from *T. lutea* were purchased from Sigma-Aldrich (St. Louis, MO, USA).

### Ethical statement

This research was approved by the Institutional Animal Care and Use Committee (IACUC) of the Korea Institute of Science and Technology (KIST): KIST No. 2020-002, Gangneung Institute, and followed the Association for Research in Vision and Ophthalmology's statement on the Use of Animals in Ophthalmic and Vision Research. In addition, we follow the Animal Research: Reporting of In Vivo Experiments (ARRIVE) recommendations. All procedures were conducted in compliance with applicable regulations and guidelines.

### Animal experiments and induction of DES model

Mice were housed in an air-conditioned animal room and maintained at 22 °C ± 2 °C, 50% ± 10% humidity, and a 12 h light/12 h dark circadian cycle. Food and water were provided ad libitum. Mice were acclimated for one week before being randomly divided into five groups of seven male 6-week-old BALB/c mice (Orientbio, Gyunggi-do, Korea). DES was induced experimentally in mice twice daily via intraperitoneal injection of 200 μL (2.5 mg/mL) of scopolamine diluted in phosphate-buffered saline (PBS; Sigma-Aldrich, St. Louis, MO, USA). *T. lutea* was administered orally to groups of mice daily at concentrations of 0 (vehicle control), 100, 150, or 300 mg/kg in 200 μL of distilled water. The control group received PBS without scopolamine. When *T. lutea* was administered orally, 200 μL distilled water was used in the control and DES group. Tear production was quantified after two weeks using a standard Schirmer’s test strip placed in the lower one-third of the temporal eyelid before closing the eye for 1 min. The strip was then removed and the length of the wet point in millimeters was measured to determine Schirmer’s test value. Staining of the corneal surface was used to determine the extent of corneal surface changes. The corneal surface was observed and scored after injecting one drop of 3% fluorescein into the inferior lateral conjunctival. Corneal staining was evaluated blindly. Mice were euthanized by cervical dislocation. The corneas were removed by flicking the eyelid of the mouse and using forceps to cut out the optic nerve from the eyeball and remove the cornea. The crystalline lens was removed from within the cornea and stored at − 80 °C. The lacrimal gland was removed from the mouse’s lower jaw using forceps, and the epidermis of the pulled area was cut away to reveal the lower half of the face. The lacrimal gland near the lower jaw was secured and removed then stored at − 80 °C protected from light.

### Histology

Corneal epidermal and lacrimal gland tissues were collected and fixed in 10% formaldehyde, followed by processing for paraffin embedding and sectioning. Sections were stained with H&E and examined at 40 × magnification using a microscope (TE-2000U, Nikon, Tokyo, Japan). the central corneal epithelial thickness was determined by dividing each cornea into five sections.

### DAB staining for immunohistochemical (IHC) analysis

Tissues from the lacrimal glands of BALB/c mice were taken and fixed with 10% formaldehyde. Fixed lacrimal gland tissues were paraffin-embedded, sectioned (5 μm), and the paraffin was removed three times for 3 min using xylene (Junsei, Tokyo, Japan). The sections were hydrated for 1 min in 100, 95, 70, and 50% ethanol. Lacrimal gland antigen was extracted by microwave with TRIS–EDTA buffer (pH 9.0). After applying 3% hydrogen peroxide to tissue slides for 15 min, the sections were blocked with 2% BSA for 1 h at room temperature. The sections were treated overnight at 4 °C with CD45 (Bio-RAD, MCA 1258GT, 1:100 dilution). After washing with PBS, the cells were treated for 2 h at room temperature with anti-rabbit IgG-HRP (Santa Cruz Biotechnology, Dallas, TX, USA, SC-2357, 1:100 dilution) and stained for 1 min with 3,3′-diaminobenzidine tetrahydrochloride (Vector Laboratories, Burlingame, CA, USA). The slides were stained for 30 s with hematoxylin and rinsed with tap water. The slices were then cover-slipped and mounted with Permount™ Mounting Medium (Thermo Fisher Scientific, Waltham, MA, USA). All slides were examined and photographed using a light microscope (Olympus, CX43, Olympus Optical Co., Tokyo, Japan). ImageJ software (version 1.53, National Institutes of Health, Bethesda, MD, USA)^48^ was used to perform quantitative data analysis.

### ARPE-19 cell culture

Human retinal epithelial ARPE-19 cells (American Type Culture Collection, ATCC, Manassas, VA, USA) were cultured in DMEM/F-12 media (Gibco, Carlsbad, CA, USA) supplemented with 10% FBS (HyClone Laboratories, Logan, UT, USA) and 1% penicillin/streptomycin (HyClone Laboratories). Cells were seeded at a density of 2 × 10^5^ per well in 6-well plates and cultured to induce an inflammatory and endoplasmic response.

### Cell viability

The effect of *T. lutea* on cell viability was evaluated by MTT assay as described previously study^49^ but with slight modifications. Cells were plated in 96-well culture plates at a density of 1.5 × 10^4^ cells/well and incubated for 24 h. Cells were then treated with various concentrations of *T. lutea* and incubated for a further 24 h. The supernatant of each well was replaced with a fresh growth medium containing 0.5 mg/mL of MTT and incubated for 1 h. The supernatant was removed and generated formazan deposits were dissolved in 200 μL of dimethyl sulfoxide. Absorbance was measured at 570 nm. Cell viability was relatively calculated as a percentage compared to negative control groups.

### Western blot analysis

Western blotting was conducted to determine cellular signaling pathways underlying the anti-inflammatory effect of *T. lutea* as described previously study^49^, but with minor modifications. Confluent ARPE-19 cells were starved in DMEM:F12 containing 1% FBS and 1% antibiotics for 24 h. Cells were then treated with *T. lutea* (50 and 100 μg/mL) for 2 h and then stimulated with 20 ng/mL of TNF-α for 15 min. The cells were rinsed three times with ice-cold DPBS and then lysed in protein extraction buffer (iNtRON Biotechnology, Gyeonggi-do, Korea) supplemented with a cocktail of protease and phosphatase inhibitors. Supernatants of lysates were obtained by centrifugation (14,000×*g*, 30 min, 4 °C) and their protein concentration was estimated using a DCTM protein assay kit (Bio-Rad, Hercules, CA, USA). Equal amounts (15–25 μg) of cellular proteins were separated by SDS-PAGE and transferred onto PVDF membranes. Membranes were incubated with 5% bovine serum albumin or skim milk, and then incubated with a 1:2000 dilution of primary antibodies (anti-phopho-ERK, anti-ERK, anti-phospho-JNK, anti-JNK, anti-phospho-p38, anti-p38, anti-phospho-AKT, anti-AKT, anti-phospho-IκB-α, anti-IκB-α, anti-phospho-p65, anti-p65, anti-α-tubulin and anti-β-action) at 4 °C and 12 h. The membranes were then rinsed with TBS-T and treated 1 h at room temperature with anti-rabbit HRP-conjugated secondary antibodies at a dilution of 1:10,000. Protein bands were detected by using SuperSignal West Femto Maximum Sensitivity Substrate (Thermo Fisher Scientific) and visualized in an iBright CL1000 gel documentation system (Thermo Fisher Scientific). The density of each protein blots was determined by using Image J software (version 1.53, NIH)^48^.

### Total PUFA extraction from *T. lutea*

Total PUFAs were extracted from *T. lutea* using the Folch technique^50^. Briefly, 100 mg of the sample was placed into a vial containing 5 mL of CH_3_Cl:MeOH (2:1, v/v) solution and ultrasonicated for 15 min. The suspension was clarified by centrifugation at 10,000 rpm for 5 min. Na_2_SO_4_ was added to the supernatant which was then filtered to eliminate any remaining water. This step was repeated two more times. The solution was then concentrated under nitrogen gas to obtain pure algal lipids.

### Fatty acid methylation

Fatty acid methylation was carried out to prepare lipid samples for gas chromatography (GC) analysis. Extracted material was added to a 10 mL vial followed by 1 mL of 0.5 N NaOH:MeOH mixture. This solution contains 400 μg/mL of internal strandard (valeric acid). The mixture was shaken for 30 s, and then incubated for 20 min at 80 °C. The mixture was then cooled at room temperature for 5 min, and then 2 mL of boron trifluoride:MeOH was added, and then agitated for 30 s. The mixture was incubated for 20 min at 80 °C followed by cooling for 10 min. A volume of 2 mL of supersaturated NaCl was added to induce phase separation and fatty acids collection. Next, 1.5 mL of *n*-hexane was added and the mixture was vortexed for 30 s before cooling at room temperature for 20 min. The layers were separated and the liquid supernatant was removed from the mixture and the water was completely removed using a saturated Na_2_SO_4_ filter.

### Identification of fatty acids

Fatty acid methyl esters (FAMEs) were examined using a GC equipped with a flame ionization detector (FID; Agilent 7890A, Agilent Technologies, Wilmington, DE, USA). Samples (1 µL) were injected into the HP-88 column of the GC (J&W 112-88A7, Agilent Technologies, Wilmington, DE, USA) (100 m × 250 μm I.D., 0.2 μm film). GC analysis was performed as follows: samples were initially held at 140 °C for 5 min, and then the temperature was ramped up at 4 °C/min to 240 °C for 15 min. The flow rate of the column was fixed at 1 mL/min. Nitrogen gas was employed as the carrier gas for the analysis, the FID was set at 280 °C, and the split ratio was 30:1. FAMEs were identified by comparing them to known standards. Each sample was measured by GC analysis in triplicate.

### Statistical analysis

All data were analyzed statistically using one-way ANOVA with Tukey’s post hoc analysis by SPSS version 18.0 (IBM Co., Armonk, NY, USA). At least three independent replicates were performed for each experiment.

## Supplementary Information

Supplementary Information.

## Data Availability

All data generated or analyzed during this study are included in this published article. Supplementary data is provided with the original article. All datasets generated and/or analyzed during this study are available from the corresponding author on reasonable request.
