# Supplementary material for: Protective effect of Tisochrysis lutea on dry eye syndrome via NF-κB inhibition
Source: Sci Rep. 2022 Nov 15;12:19576. doi: 10.1038/s41598-022-23545-7 (PMC9666437; doi:10.1038/s41598-022-23545-7)
Supplement: Supplementary file 1 — Supplementary Information. [file 41598_2022_23545_MOESM1_ESM.docx]

**Supplementary Data S1-S5**


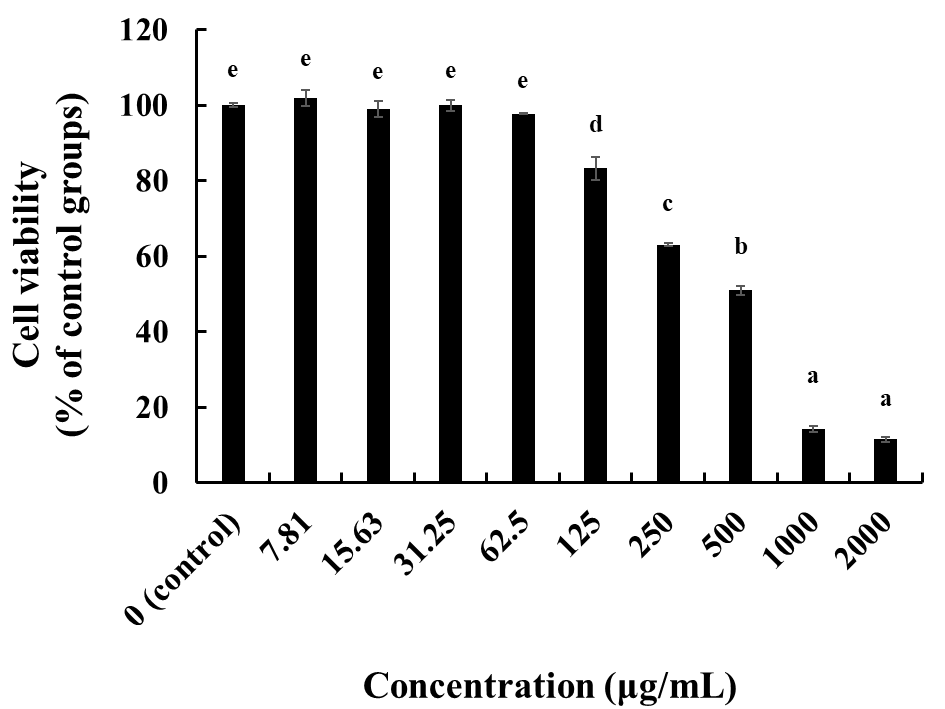


**Figure S1. Cytotoxicity of different concentrations of *T. lutea* in ARPE-19 cells.**

**Figure S2. Immunohistochemical staining results of lacrimal gland tissues.** (A) IHC images using an anti-CD45 antibody related to the immune system of lacrimal gland tissues at different concentrations of *T. lutea*. (B) Quantity of IHC-stained lacrimal gland tissues by ImageJ. ^***^ *p* < 0.001 versus negative control. Data were analyzed statistically using one-way ANOVA followed by Tukey’s post hoc test.


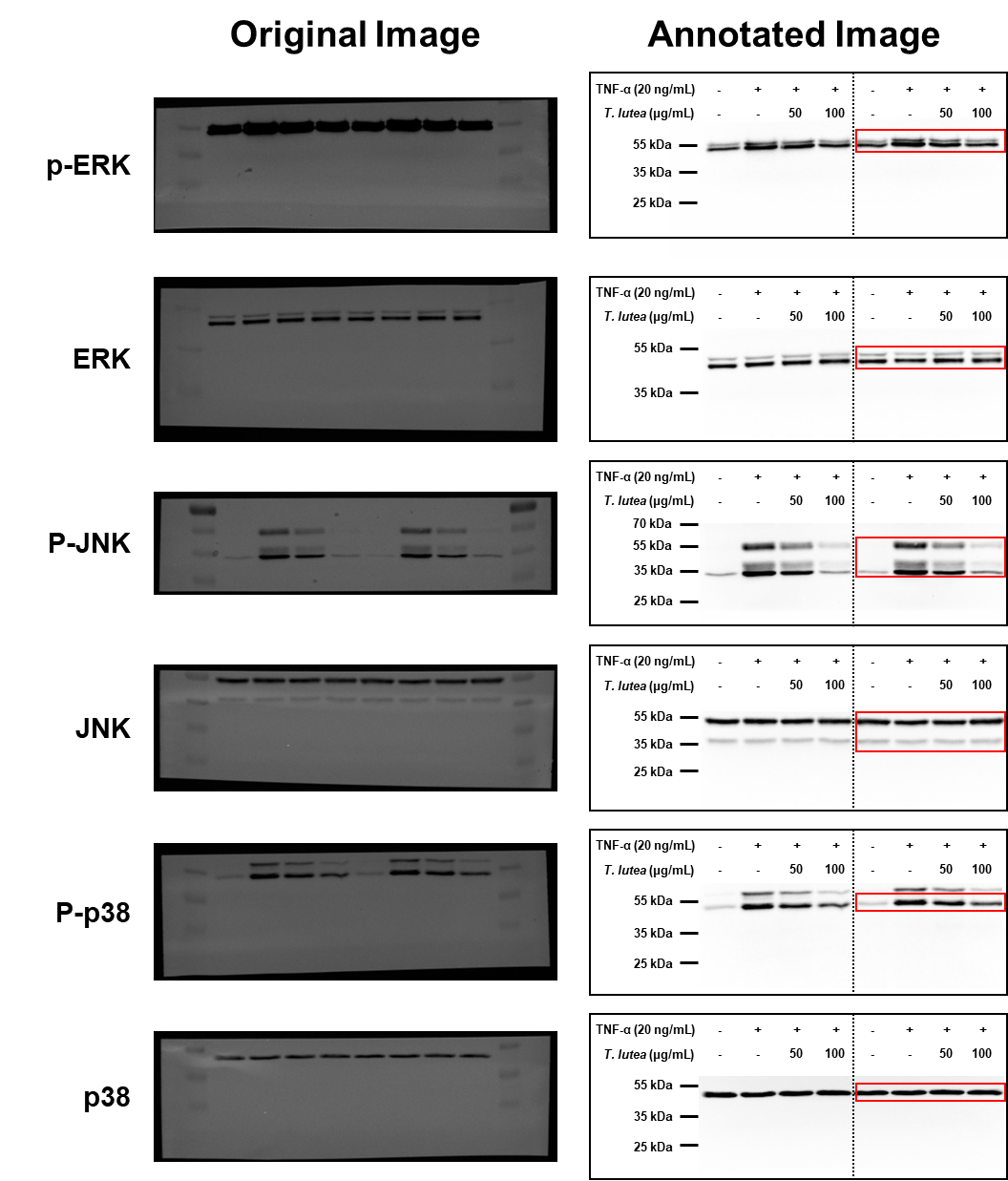


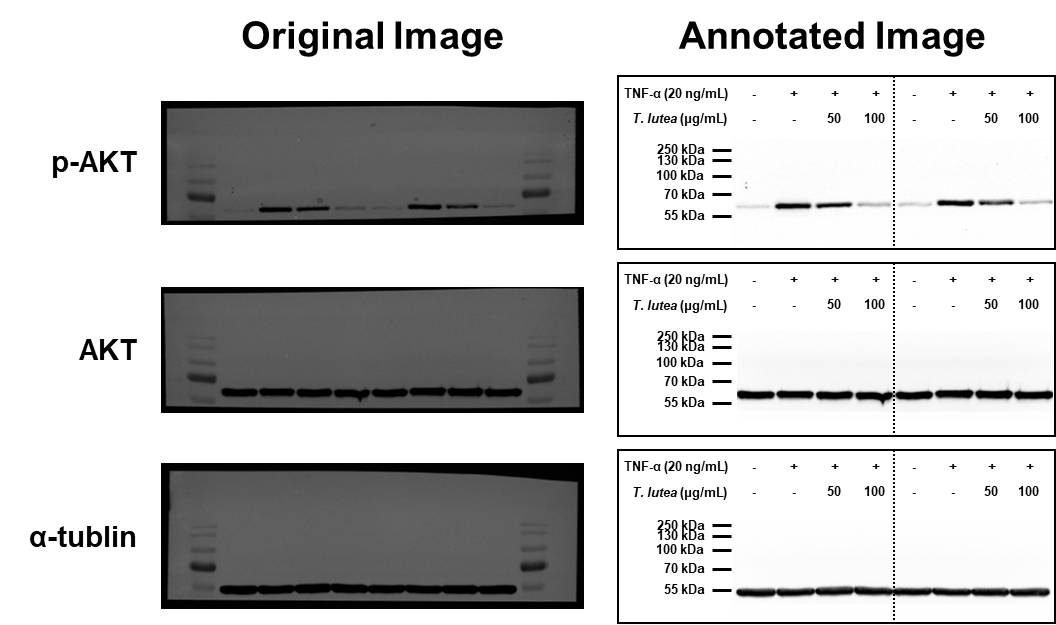


**Figure S3. Western blot raw data from Figure 3.** The left side displays the original data, while the right shows the equivalent protein quantification section based on the molecular weight of the original image.


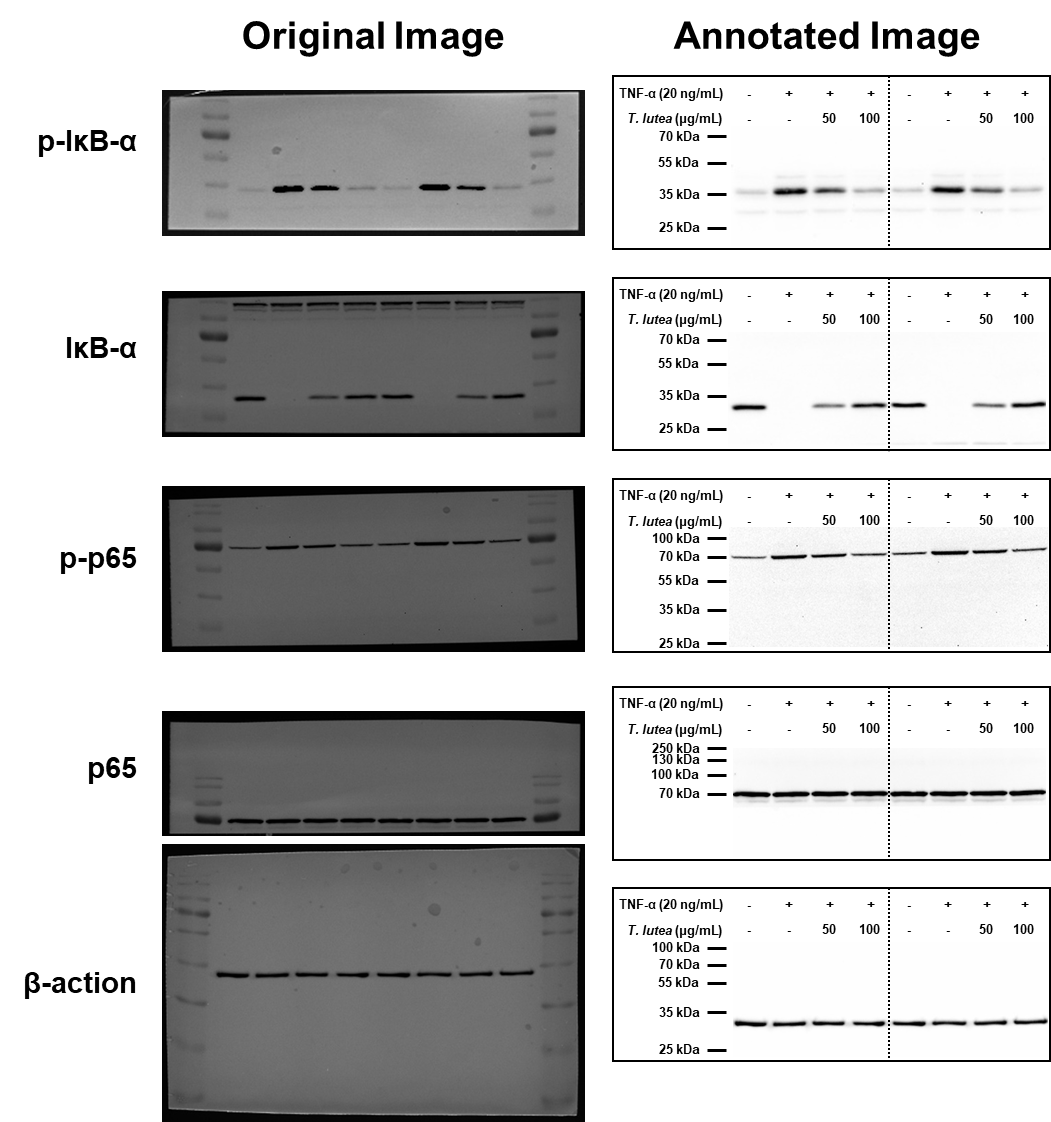


**Figure S4. Western blot raw data from Figure 4.** The left side displays the original data, while the right shows the equivalent protein quantification section based on the molecular weight of the original image.


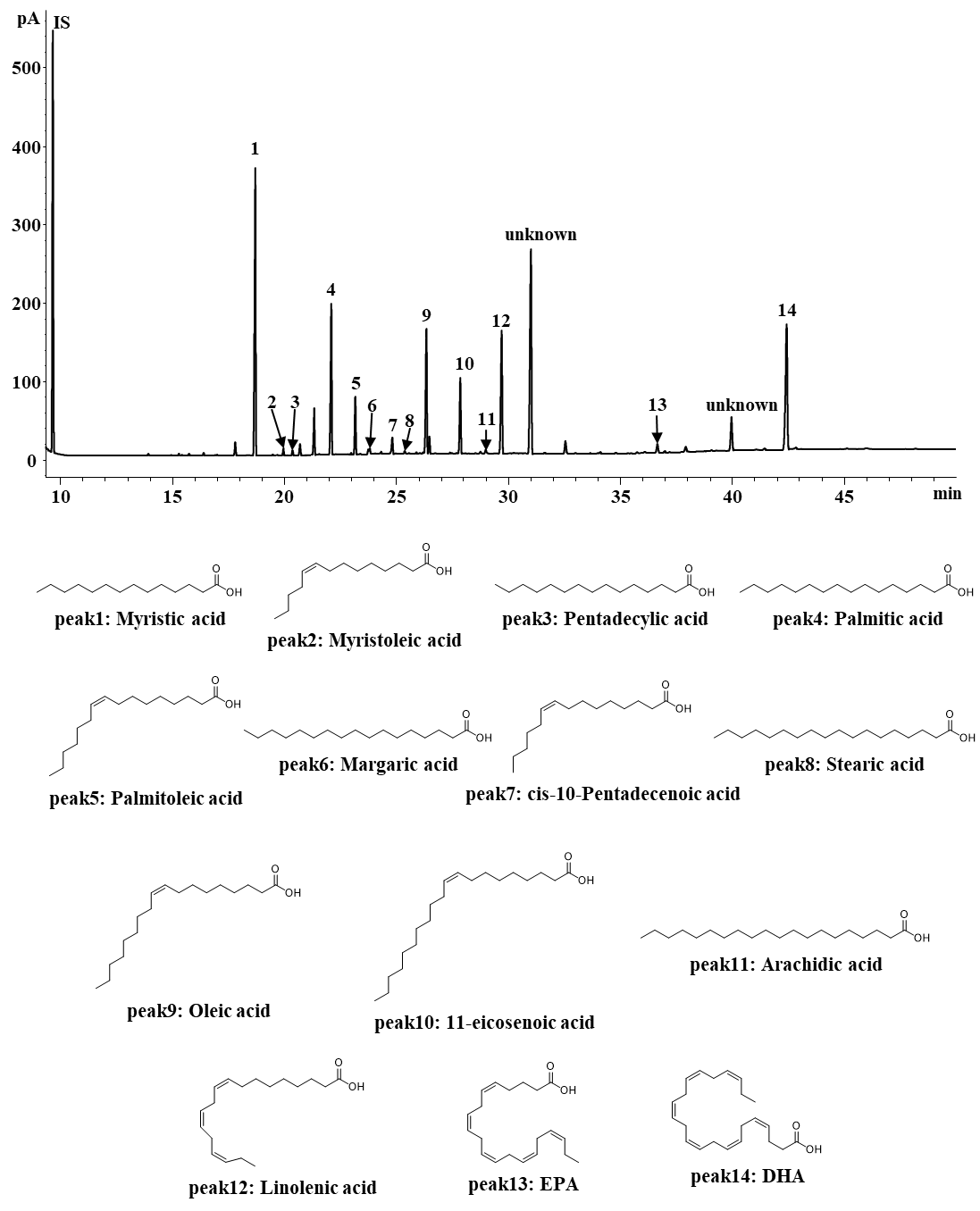


**Figure S5. GC-FID spectra for PUFAs from *T. lutea.***
